# Supplementary material for: Bridging the research gap in conflict-affected countries: A multi-institutional study on medical students’ research involvement and barriers in Yemen
Source: PLoS One. 2026 Apr 29;21(4):e0348188. doi: 10.1371/journal.pone.0348188 (PMC13127913; doi:10.1371/journal.pone.0348188)
Supplement: S1 File — Knowledge assessment questionnaire. This file contains the full set of knowledge assessment items used in the study to evaluate content validity, along with the attitude and perceived barriers scales included in the survey instrument. (DOCX) [file pone.0348188.s001.docx]

**Research knowledge assessment questionnaire**

The following ten multiple-choice questions were used to assess undergraduate medical students’ knowledge of basic research concepts. Each question had one correct answer. One point was awarded for each correct response, resulting in a total possible score ranging from 0 to 10.

**Question 1. How would you define a scientific hypothesis?**

A. An answer or solution to a question which has the capacity of verification or empirical demonstration
B. An answer or solution to a question
C. A proposed idea or thought
D. Logical deduction of premises that may or may not be verified empirically

**Correct answer:** A

**Question 2. How would you define a scientific theory?**

A. Speculation or assumption with no or insufficient evidence
B. A system of hypotheses logically connected to one another, with a common background, some of which have been verified
C. Scientific hypotheses that may be proven but lack evidence for verification
D. A set of scientific knowledge on a given topic or area

**Correct answer:** B

**Question 3. How would you define scientific truth?**

A. The truth that will be reached through scientific research
B. Absolute truth
C. Consensus of competent experts
D. Facts that can be found in textbooks

**Correct answer:** A

**Question 4. The essential characteristic of science is:**

A. All scientific conclusions are temporary
B. Scientific theory must influence natural phenomena
C. Rather obvious scientific conclusions do not have to be testable
D. An experiment is not an objective model of nature

**Correct answer:** A

**Question 5. A scale from 1 to 5 (such as examination grades) is called:**

A. Ratio scale
B. Nominal scale
C. Ordinal scale
D. Interval scale

**Correct answer:** C

**Question 6. Representativeness is a key characteristic of a:**

A. Population
B. Professional paper
C. Scientific research
D. Sample

**Correct answer:** D

**Question 7. MEDLINE is:**

A. The first and best-known online medical journal
B. An international association of medical informaticians
C. Printed form of Excerpta Medica
D. A medical bibliographic database

**Correct answer:** D

**Question 8. To check the number of citations your published paper has received, the best source to search is:**

A. Author index of the MEDLINE database
B. Author index of the Science Citation Index database
C. Author index of the Current Contents database
D. Citation index of the Science Citation Index database

**Correct answer:** D

**Question 9. Which of the following is a part of a scientific paper?**

A. Author’s curriculum vitae
B. Letter to the editor enclosed with the paper
C. Professional paper
D. Acknowledgment to persons who assisted during the research

**Correct answer:** D

**Question 10. All the following rules apply to writing the Introduction section of a scientific paper EXCEPT:**

A. Clearly state why the research was started
B. Do not explain textbook facts
C. Do not explain words from the title of the paper
D. Make it longer rather than shorter

**Correct answer:** D

**Scoring**

- Each correct answer = 1 point
- Total possible score = 10
- Higher scores indicate greater research knowledge
