# Supplementary material for: Bridging the research gap in conflict-affected countries: A multi-institutional study on medical students’ research involvement and barriers in Yemen
Source: PLoS One. 2026 Apr 29;21(4):e0348188. doi: 10.1371/journal.pone.0348188 (PMC13127913; doi:10.1371/journal.pone.0348188)
Supplement: Attachment — Submitted filename: Point to point response.pdf [file pone.0348188.s003.pdf]

Response to reviewers

We sincerely thank the editor and reviewers for their constructive and insightful comments, which have significantly improved the quality, clarity, and rigor of our manuscript. We have carefully addressed all comments and revised the manuscript accordingly. All changes have been incorporated into the revised version of the manuscript. Detailed point-by-point responses are provided below.

Reviewer #1

Comment 1:

This manuscript represents a valuable and much-needed contribution to the fields of medical education and global health. It provides crucial baseline data from a challenging context. The methodological strengths are evident in the national scope and sample size. To fully realize its potential, the authors must address the key issues of the contradictory data availability statement, the misinterpretation of the low R-squared values, the typographical errors in tables, and the need for more strategic citations to frame the conflict context.

Response:

We sincerely thank the reviewer for the positive assessment of the manuscript and for highlighting its relevance and methodological strengths. We have carefully addressed all the issues raised:

- **Data availability:** The data availability statement has been revised for clarity and consistency. The dataset is now publicly available through Figshare, and the following statement has been added to the manuscript:
  "The datasets generated and/or analyzed during this study are publicly available in the Figshare repository at https://doi.org/10.6084/m9.figshare.30927329."
- **Interpretation of low R-squared values:** We revised the Results and Discussion sections to explicitly acknowledge the low R^2 values and to clarify that, although certain predictors were statistically significant, they explain only a small proportion of the variance. This limitation is now clearly stated and discussed to avoid overinterpretation.

- **Typographical errors in tables:** All tables were carefully reviewed and corrected. This included resolving duplicated attitude items, correcting mislabeled variables, standardizing formatting, and improving table titles and footnotes.
- **Conflict-context framing:** We strengthened the Discussion by adding targeted citations and explicitly linking the identified research barriers to the impact of ongoing conflict on higher education, including faculty shortages, damaged infrastructure, limited funding, and restricted access to academic resources.

Reviewer #2

Comment 1 (In abstract):

A total of 1,387 students completed a ………… this need to be in the results section.

Response:

We thank the reviewer for this helpful suggestion. The abstract has been revised so that the number of participants is now reported exclusively in the Results section of the abstract, in line with standard reporting conventions.

Comment 2 (In abstract):

I suggest removing the word 'national,' as the manuscript does not present information about the geographical distribution or representativeness of the ten included universities.

Response:

We agree with this comment. The term "national" has been removed throughout the manuscript and replaced with "multi-institutional" where appropriate, including in the title, abstract, and main text.

Comment 3 (In abstract):

Furthermore, the data collection period ('between November and December 2024') should be omitted from the abstract to enhance clarity and brevity.

Response:

This suggestion has been implemented. The data collection period has been removed from the abstract and is now reported only in the Methods section.

Comment 4 (In abstract):

The reported 20.5% publication rate appears higher than expected for undergraduate medical students. It may be helpful to double-check the data or clarify the criteria used for defining 'published research paper'.

Response:

We appreciate this observation. The data were rechecked and confirmed. To improve clarity, we have clarified in the Methods section that "publication" refers to authorship on a research paper published in a peer-reviewed journal, including supervised student publications. Additional contextual clarification has also been added in the Discussion.

<u>In Introduction</u>

Comment 5:

"Health policies" instead of "health policy".

Response:

The wording has been corrected to "health policy."

Comment 6:

Combine the first two sentences more coherently.

Response:

The sentences were merged and rephrased for improved coherence and flow, as suggested.

Comment 7:

Replace "untapped" with "unrealized".

Response:

This wording change has been implemented.

Comment 8:

Claims about the Arab region require stronger citations.

Response:

We added additional, relevant citations from the Arab region to appropriately support this statement.

Comment 9:

Change "remain low" to "remain limited".

Response:

The wording has been revised accordingly.

Comment 10:

Change "national level understanding" to "comprehensive understanding" and "addresses that need" to "addresses this gap".

Response:

These wording changes have been incorporated, and the paragraph was revised to improve clarity and precision.

Comment 11:

Clearly define the study objectives and include a research question.

Response:

The Introduction now explicitly states the study objectives and includes a clear research question guiding the investigation.

In Methods

Comment 12:

Remove "nationwide" and use "multi-institutional".

Response:

The term "nationwide" has been removed and replaced with "multi-institutional" throughout the Methods section.

Comments 13 and 14:

Clarify the population estimate and sample size calculation.
Design effect is not appropriate for convenience sampling.

Response:

We thank the reviewer for these important methodological comments. In response, the sample size determination section has been substantially revised to improve clarity and methodological rigor. Rather than relying on an uncertain national population estimate, the revised calculation is now based on the primary outcome of interest, namely the mean research knowledge score, using parameters derived from a previously published regional study among undergraduate medical students. This approach aligns with recommended practices for cross-sectional studies assessing continuous outcomes.

Additionally, we fully agree that the application of a design effect is not appropriate in the context of convenience sampling. Therefore, all references to a design effect have been removed, and the sampling approach and sample size calculation have been fully aligned and clarified. The revised Methods section now transparently reports a minimum required sample size of 370 participants, which was exceeded in the final sample.

Comment 15:

Actual number of respondents should be reported in Results, not Methods.

Response:

This has been corrected. The achieved sample size is now reported only in the Results section.

Comment 16:

Convenience sampling should be acknowledged as a limitation.

Response:

This limitation is now explicitly stated and discussed in the Limitations section.

Comment 17:

Clarify whether the questionnaire was piloted.

Response:

The Methods section has been updated to clarify that the questionnaire was reviewed and piloted among a small group of medical students to ensure clarity and contextual appropriateness.

Comment 18:

Provide the full set of knowledge assessment items.

Response:

We have addressed this important point by including the complete set of ten research knowledge multiple-choice questions as **Supplementary material 1**, with clearly defined correct answers and scoring methodology.

<u>In Discussion</u>

Comment 19:

Strengthen the discussion by explicitly linking barriers to the conflict-affected context.

Response:

The Discussion has been substantially revised to more explicitly connect identified barriers, such as lack of mentorship, limited infrastructure, and restricted access to academic resources, to the ongoing conflict and its impact on Yemen's higher education system.
