# Supplementary material for: Bridging the research gap in conflict-affected countries: A multi-institutional study on medical students’ research involvement and barriers in Yemen
Source: PLoS One. 2026 Apr 29;21(4):e0348188. doi: 10.1371/journal.pone.0348188 (PMC13127913; doi:10.1371/journal.pone.0348188)
Supplement: Attachment — Submitted filename: barriers in Yemen Review Report.docx [file pone.0348188.s002.docx]

**Dear Editor and Authors,**

Thank you for the opportunity to review this timely and important manuscript. This national study addresses a critical gap in our understanding of research capacity among medical students in a severely under-researched, conflict-affected setting. The topic is highly relevant to global health equity, the sample size is substantial and national in scope, and the manuscript is generally well-structured. However, several key methodological and presentational issues require attention to strengthen the manuscript's validity, clarity, and overall impact.

**-** The abstract provides a concise and accurate summary of the study's objectives, methods, key findings, and conclusions. The stark contrast between positive attitudes (79.8%) and low publication rates (20.5%) is effectively communicated. To fully convey the strength of the analytical findings, the abstract should highlight the **low R-squared values from the regression analysis** (e.g., R² = 0.01 for knowledge). This is a fundamental note, indicating that while certain factors are statistically significant predictors, they explain only a small portion of the variance, pointing to the overwhelming influence of unmeasured systemic barriers.

**-** The background effectively establishes the global research disparity and the importance of undergraduate research engagement. The rationale for focusing on Yemen, given its conflict and humanitarian crisis, is compelling and well-justified. The transition from general barriers to the specific gap in Yemeni literature is logical. To further strengthen the framing, the statement, **"Given Yemen's ongoing conflict... a national level understanding... is urgently needed,"** could be positioned more prominently at the end of the introduction to powerfully frame the study's unique contribution and necessity.

**-** The methodology is strong for a cross-sectional survey and is generally well-reported.

*****The national cross-sectional design is appropriate. The use of a **convenience sampling** technique is a significant limitation that is acknowledged but warrants a more detailed discussion in the limitations section regarding the potential for selection bias, particularly the overrepresentation of male students (70.7%), which may limit the generalizability of the findings.

*The a priori sample size calculation using the Raosoft calculator and the application of a design effect are clear strengths.

*****The use of a previously validated questionnaire is a strength. However, the manuscript would be significantly strengthened by a **brief description of the domains covered in the 10 knowledge questions** (e.g., research ethics, study designs, biostatistics). Providing the full questionnaire as a supplementary file is highly recommended to enhance transparency and reproducibility.

*****The statistical plan is appropriate and well-detailed. The mention of a post-hoc test for ANOVA is cut off ("Where significant differences were identified through ANOVA."). This sentence should be completed (e.g., "... a Tukey HSD test was applied to identify between-group differences.").

**-**The results are data-rich and clearly presented in the tables.

*****There are no major terminology errors on the scale of "hardiness/hardness," but clarity is needed. In Table 3, the heading for the attitude score for "Academic score" reads **"total attitude (2.9 ± 1.8) and total attitude (24.8 ± 2.7)"**. The first one is clearly a typo and should be **"total knowledge".**

*****The regression analysis (Table 4) is a key result. The authors should explicitly comment on the **very low R-squared values** (0.004 to 0.01) in the text. This is critical for a correct interpretation, indicating that the model explains very little variance and that factors beyond age, academic year, and GPA are the primary drivers of knowledge and attitudes.

**-Table 2:** This table contains a **duplication error**, with two identical items listed as "Research will not help in better understanding of subject" (items 2 and 7). One of these is likely a different statement from the original questionnaire and must be corrected.

**-** The discussion does a good job of contextualizing the findings within the existing literature from similar settings and highlighting the systemic barriers.

*The paragraph on **"Psychological readiness" and "cognitive flexibility"** is interesting but feels somewhat speculative and disconnected, as these constructs were not measured in the present study. This section should be either more carefully integrated as a theoretical suggestion for future research or condensed to maintain a sharper focus on the empirical findings.

*The **clinical and educational implications** are relevant but could be more specific. Instead of "targeted interventions," suggest concrete actions based on the top barriers, such as "integrating mandatory, practical research methodology workshops into the core curriculum," "establishing a formal faculty-student mentorship program for research," and "advocating for institutional policies that provide access to online libraries and research software."

*The discussion of the **conflict-affected context** could be deepened. While mentioned, a more explicit discussion of how the specific realities of the Yemeni conflict (e.g., infrastructure collapse, internet blackouts, brain drain of faculty) directly exacerbate the identified barriers would powerfully underscore the unique challenges faced in this setting.

**-** The reference list is comprehensive. The manuscript can be significantly strengthened by incorporating several recent, highly relevant studies that provide a stronger regional and conflict-sensitive context.

-In the **introduction or background**, citing a study on research barriers in a similar context, such as **Abusamak et al. (2024) from Jordan**, would immediately ground the study in the regional literature.

-In the **discussion**, when emphasizing the impact of the conflict, the narrative review by **on the mental health impact of war in Gaza (EMHJ)** provides a powerful macro-level context, suggesting that the observed barriers and stress are compounded by pre-existing, widespread trauma and system collapse.

-Furthermore, when discussing the stress and additional burdens on students, the study by **on the stress and resilience of nursing students during political violence (PLoS ONE)** offers a compelling parallel. It supports the idea that the academic environment in conflict zones is uniquely stressful, which directly competes with the cognitive and temporal resources needed for research engagement.

**In conclusion,** this manuscript represents a valuable and much-needed contribution to the fields of medical education and global health. It provides crucial baseline data from a challenging context. The methodological strengths are evident in the national scope and sample size. To fully realize its potential, the authors must address the key issues of the **contradictory data availability statement, the misinterpretation of the low R-squared values, the typographical errors in tables,** and the need for **more strategic citations** to frame the conflict context. With these revisions, this manuscript will be significantly strengthened and will make a strong candidate for publication.
