# Supplementary material for: Bridging the research gap in conflict-affected countries: A multi-institutional study on medical students’ research involvement and barriers in Yemen
Source: PLoS One. 2026 Apr 29;21(4):e0348188. doi: 10.1371/journal.pone.0348188 (PMC13127913; doi:10.1371/journal.pone.0348188)
Supplement: Attachment — Submitted filename: Point to point response.docx [file pone.0348188.s004.docx]

**Response to Reviewer #2**

We sincerely thank the reviewer for the constructive and insightful comments that helped improve the clarity and methodological rigor of our manuscript. We have carefully revised the manuscript in accordance with the suggestions provided. All changes have been incorporated into the revised manuscript.

**A) Abstract**

**Comment 1**

"A cross-sectional, multi-institutional survey was conducted among undergraduate medical students across ten universities in Yemen."
"A validated online questionnaire was used to collect data on demographic characteristics, prior research experience, attitudes toward research, research knowledge, and perceived institutional barriers."
This is a further detailed and repeated statement which was previously stated earlier in the introduction.

**Response:**
Thank you for this observation. The Methods section of the abstract has been revised to reduce redundancy and improve conciseness. The description of the questionnaire has been simplified while retaining the essential methodological details.

**Comment 2**

"Medical students in Yemen demonstrate generally positive attitudes toward research" this needs to be revised since the study did not represent all.

**Response:**
We agree with the reviewer and have revised this statement to avoid overgeneralization. The sentence has been modified to:
*"Participating medical students demonstrated generally positive attitudes toward research."*

**Comment 3**

Over interpretation has been noticed in different sections in the manuscript this needs to be revised.

**Response:**
Thank you for highlighting this point. We carefully reviewed the manuscript and revised several sentences throughout the abstract, discussion, and conclusion to ensure more cautious interpretation of the findings. Language suggesting causality or generalization has been replaced with more neutral phrasing (e.g., “suggest,” “may indicate,” and “among the surveyed students”).

**Comment 4**

Keywords: Please revise the keywords to make them more professional.

**Response:**
The keywords have been revised to improve clarity and relevance. The updated keywords are:
*Undergraduate medical education; Research engagement; Medical students; Research barriers; Conflict-affected settings; Yemen.*

**B) Background**

**Comment 1**

Authors may further sharpen the focus by more explicitly linking global disparities in research output to medical education and undergraduate research training, rather than research capacity in general.

**Response:**
We appreciate this suggestion. Additional sentences were added to the background section to explicitly connect global disparities in research output with the role of undergraduate medical education and research training in strengthening research capacity in low-resource settings.

**Comment 2**

Avoiding slightly rhetorical / generic phrases such as “not merely academic, strikingly uneven and perpetuates.....” and using more neutral language.

**Response:**
Thank you for this comment. The background section was carefully revised to remove rhetorical language and replace it with more neutral scientific wording. For example, the phrase referring to disparities being “not merely academic” was revised to a more neutral description of the potential implications for evidence-based decision-making.

**Comment 3**

The transition between the first and second also third paragraphs could be smoother by briefly signaling the shift from regional evidence to the Yemeni context.

**Response:**
We have revised the transitions between paragraphs to improve logical flow. Additional linking sentences were introduced to clearly move from the global perspective to regional evidence and finally to the specific context of Yemen.

**Comment 4**

Ensure consistent use of terms such as research engagement, research participation, and research involvement throughout the background to avoid conceptual ambiguity.

**Response:**
The manuscript has been revised to improve consistency in terminology. The term **“research engagement”** is now used consistently throughout the manuscript to describe students’ participation in research-related activities.

**Comment 5**

The background would benefit from a clearer indication of how the findings could inform educational policy, curriculum development, or institutional strategies in Yemen.

**Response:**
We agree with the reviewer and have added sentences in the background section emphasizing how the findings of this study may inform curriculum development, educational strategies, and institutional policies aimed at strengthening undergraduate research engagement in Yemen.

**C) Methods**

**Comment 1**

Manuscripts should adhere to Observational studies criteria STROBE.

**Response:**
The manuscript has been revised to better align with the **STROBE guidelines for cross-sectional studies**. Relevant methodological details have been clarified, and the STROBE checklist has been prepared for submission with the revised manuscript.

**Comment 2**

"This study employed a cross-sectional analytical design and was conducted among undergraduate medical students from ten universities in Yemen." What do you mean by "analytical"?

**Response:**
Thank you for this comment. To improve clarity, the term **“analytical”** has been removed. The sentence has been revised to state that the study employed a **cross-sectional study design**.

**Comment 3**

No details were stated regarding those "ten universities".

**Response:**
Additional information has been added in the Methods section indicating that the participating institutions included both public and private universities located in different regions of Yemen.

**Comment 4**

"...the survey was disseminated broadly, yielding a final sample of 1,387 respondents." No need to be mentioned in this section.

**Response:**
We agree with the reviewer. The statement referring to the final sample size has been removed from the sample size subsection and is now presented only in the Results section.

**Comment 5**

"Data collection was conducted using an online survey administered during the study period." Need for wording, further authors should indicate the online platform.

**Response:**
The Methods section has been revised to clarify the data collection procedure and specify the platform used. The revised text now indicates that the survey was administered electronically using **Google Forms**.

**Comment 6**

The term “primary outcome of interest” should be clearly defined.

**Response:**
We have clarified that the **primary outcome used for the sample size calculation was the mean research knowledge score among undergraduate medical students**.

**Comment 7**

The sample size calculation relies on data from a Jordanian population.

**Response:**
We have clarified this point in the Methods section and added a statement explaining that estimates from the Jordanian study were used because no prior large-scale studies assessing research knowledge among Yemeni medical students were available.

**Comment 8**

"Cronbach’s alpha values ranging from 0.70 to 0.80 across relevant sections." Authors need clarify for each section.

**Response:**
Thank you for this suggestion. The internal consistency values are now reported separately for each scale. The revised manuscript reports **Cronbach’s alpha values of 0.78 for the attitude scale and 0.81 for the perceived barriers scale**.

**Comment 9**

In the abstract authors have stated linear regression but no information in methods.

**Response:**
We appreciate this comment. The statistical analysis section has been revised to explicitly describe the use of **multiple linear regression analyses** to identify factors associated with research knowledge and attitude scores.

**Comment 10**

Did the authors perform normality test?

**Response:**
Yes. We have now added a statement in the statistical analysis section indicating that **normality of continuous variables was assessed using the Shapiro–Wilk test and visual inspection of histograms** prior to conducting parametric analyses.

**Comment 11**

In the results section there were missing data in Table 3 in term of comparison of total knowledge and total attitude scores by question stating "Have you ever published your research paper in journals?"

**Response:**
Thank you for identifying this omission. The variable **“Have you ever published your research paper in journals?”** has now been added to Table 3, and the corresponding statistical comparison has been included in the Results section.

**Comment 12**

In the discussion section authors need to avoid rhetorical / generic phrases and use more neutral language.

**Response:**
The Discussion section has been carefully revised to reduce rhetorical language and ensure a more neutral scientific tone. Several sentences were modified to avoid overinterpretation and to present the findings in a more cautious and evidence-based manner.
